# Loss of N-Glycanase 1 Alters Transcriptional and Translational Regulation in K562 Cell Lines

**DOI:** 10.1534/g3.119.401031

**Published:** 2020-04-02

**Authors:** William F. Mueller, Petra Jakob, Han Sun, Sandra Clauder-Münster, Sonja Ghidelli-Disse, Diana Ordonez, Markus Boesche, Marcus Bantscheff, Paul Collier, Bettina Haase, Vladimir Benes, Malte Paulsen, Peter Sehr, Joe Lewis, Gerard Drewes, Lars M. Steinmetz

**Affiliations:** * European Molecular Biology Labs, Genome Biology Unit, Heidelberg, Germany, Meyerhofstrasse 1, Heidelberg, Germany, 69117; † Stanford University, CA, 94305; ‡ Cellzome GmbH, a GlaxoSmithKline Company, Meyerhofstrasse 1, Heidelberg, Germany, 69117

**Keywords:** autophagy, deglycosylation, NFE2L1, NGLY1deficiency, NRF1

## Abstract

N-Glycanase 1 (NGLY1) deficiency is an ultra-rare, complex and devastating neuromuscular disease. Patients display multi-organ symptoms including developmental delays, movement disorders, seizures, constipation and lack of tear production. NGLY1 is a deglycosylating protein involved in the degradation of misfolded proteins retrotranslocated from the endoplasmic reticulum (ER). NGLY1-deficient cells have been reported to exhibit decreased deglycosylation activity and an increased sensitivity to proteasome inhibitors. We show that the loss of NGLY1 causes substantial changes in the RNA and protein landscape of K562 cells and results in downregulation of proteasomal subunits, consistent with its processing of the transcription factor NFE2L1. We employed the CMap database to predict compounds that can modulate NGLY1 activity. Utilizing our robust K562 screening system, we demonstrate that the compound NVP-BEZ235 (Dactosilib) promotes degradation of NGLY1-dependent substrates, concurrent with increased autophagic flux, suggesting that stimulating autophagy may assist in clearing aberrant substrates during NGLY1 deficiency.

N-glycanase 1 (NGLY1) is a highly conserved deglycosylase known to function as part of the endoplasmic reticulum associated degradation (ERAD) pathway, facilitating protein surveillance and the clearance of misfolded proteins (Suzuki *et al.* 2016). A lack of NGLY1 function leads to improper processing of ERAD substrates and is hypothesized to result in the aggregation of partially glycosylated, partially degraded intermediates, however this has not been shown in a human cell line (Huang *et al.* 2015). Under normal conditions, deglycosylation of N-linked asparagine residues is accompanied by their conversion to aspartic acid by cleaving a bond between the be ta-aspartyl glycosamine linkage and the amino acid side chain (Suzuki *et al.* 2016). In 2012, a patient was first described with a mutation in the *NGLY1* gene (Need *et al.* 2012). Multiple similar patients have since been described, establishing NGLY1 deficiency as a monogenic loss-of-function rare disease (Need *et al.* 2012; Enns *et al.* 2014; Caglayan *et al.* 2015; Heeley and Shinawi 2015; Lam *et al.* 2017; van Keulen *et al.* 2019).

The NGLY1-mediated amino acid conversion via deglycosylation can act as a protein processing step for multiple factors. For example, it facilitates the conserved maturation process of the NFE2L1 transcription factor. NGLY1 also acts on the degradation of ER resident proteins like the sterol biosynthesis factor HMGR (Leichner *et al.* 2009; Koizumi *et al.* 2016; Lehrbach and Ruvkun 2016; Tomlin *et al.* 2017; Lehrbach *et al.* 2019). NFE2L1 is a necessary proteotoxic stress sensor, triggering proteasome subunit transcription as part of the proteasome bounce-back response (Radhakrishnan *et al.* 2010). It has also been shown to trigger glutathione metabolism when cells are under oxidative stress (Kong *et al.* 2018). Most recently, NGLY1 deficiency was shown to adversely affect mitochondrial function and biogenesis through an unexplained mechanism that also could result in an increase in cytokine signaling (Kong *et al.* 2018; Yang *et al.* 2018).

The body of data around NGLY1 has resulted in proof-of-concept experiments for two possible therapeutic avenues to treat patients with NGLY1 deficiency. The first possible treatment option for some clinical phenotypes may be the inhibition of endo-N-acetylglucosaminidase (ENGase). ENGase is another cytosolic enzyme that hydrolyzes glycans, but canonically acts downstream of NGLY1 after removal of the glycans from the peptide (Suzuki *et al.* 2002). An increase in accumulation of NGLY1-dependent substrates in an NGLY1-deficient cell line was rescued by the knockdown (KD) of ENGase in the same line (Huang *et al.* 2015). This suggests that the partially deglycosylated peptides processed by ENGase are more toxic than the fully glycosylated peptides remaining when neither ENGase and NGLY1 are present. In support of this, a double ENGase-NGLY1 KO approach was found to rescue lethality of NGLY1 KO mice on an organismal level, but did not alleviate all mouse phenotypes (Fujihira *et al.* 2017).

The second possible therapy for NGLY1 deficiency involves NFE2L1 and related factors or pathways. NFE2L1 is closely related to NFE2L2, a transcription factor that regulates oxidative stress pathways and also activates proteasomal transcription (Kwak *et al.* 2003). Activation of NFE2L2 is another possible treatment option and has been shown to rescue small body size phenotypes in fly larva and worm models of NGLY1 deficiency (Iyer *et al.* 2019). While multiple compounds have been found that modulate NFE2L2 activity, NFE2L2-targeted therapy would have to overcome significant clinical hurdles and be very carefully managed as mutations that cause constitutive activation of NFE2L2 also contribute to disease (Cuadrado *et al.* 2019; Huppke *et al.* 2017). While both therapeutic avenues have experimental evidence, it is unclear if a single treatment will ameliorate the downstream effects of both; clearing accumulated proteins may not rescue NFE2L1 processing and alternative activation of NFE2L1 target genes may not clear accumulated proteins. Due to a lack of human derived experimental systems, it is also unclear what the full extent of the downstream effects of NGLY1 loss are.

To learn more about NGLY1 deficiency in a human cell model, we edited the *NGLY1* gene in a human myelogenous leukemia cell line, K562. We performed whole transcriptome, whole proteome, and thermal proteome profiling (TPP) analysis on K562 cells lacking NGLY1 to determine the genes and pathways influenced by its loss of function (Figure 1, Supplementary figures EV1 and EV2) (Franken *et al.* 2015). We show that our K562 cell model faithfully replicates previously discovered molecular phenotypes related to NFE2L1 signaling. Multiple genes related to protein aggregation are differentially regulated on both the protein and transcript level. The expression changes in K562s cells correlate with an increase in protein aggregation. Using the identified set of differentially expressed genes in K562 cell lines, we queried a comparative transcriptome drug-expression database to identify compounds that similarly or oppositely alter the transcriptome (Lamb *et al.* 2006). Using these identified compounds, we show that the K562 NGLY1-deficient system we established is functional for high-throughput FACS screening. From the results of that screen, we identified Dactosilib (NVP-BEZ235), a dual mTOR/PI3K inhibitor that increases the degradation of NGLY1 substrate proteins likely through an increase in autophagic flux (Maira *et al.* 2008). We also identified PAC-1, a zinc chelating caspase inhibitor possibly acting on NGLY1 through competitive chelation of zinc (Putt *et al.* 2006).

**Figure 1:**
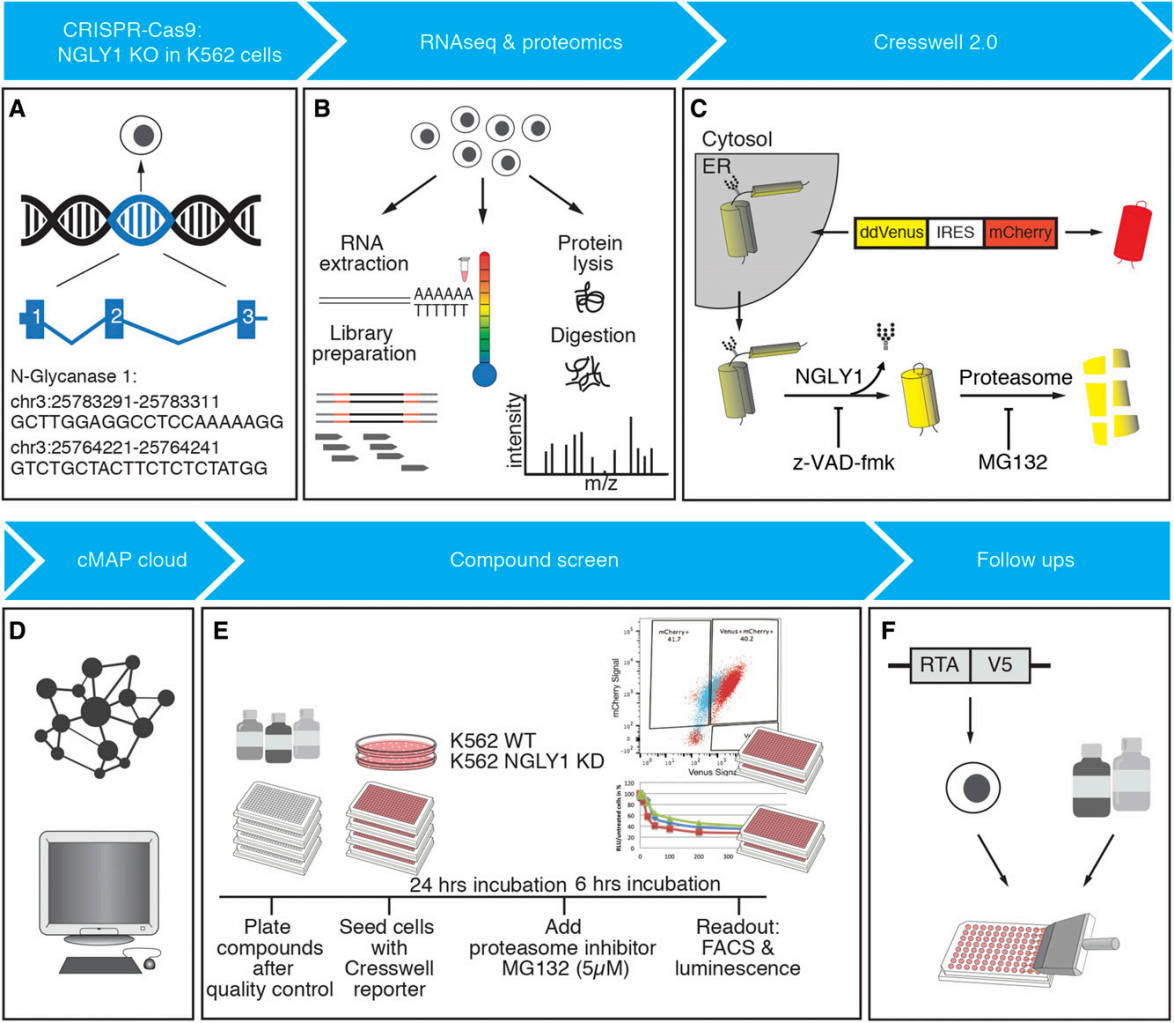
Workflow for the application of multi-omic data to drug screening in NGLY1 KD K562 cell lines.

## Materials and Methods

K562 cells were transfected with two single guide RNAs (sgRNAs) targeting exon 1 and 3 (p306_gRNA Puro, GFP) of NGLY1 and Cas9 plasmid (lentiCas9-Blast) by Nucleofection according to the manufacturer’s protocol (Nucleofector, Lonza) (Morgens *et al.* 2017). LentiGuide-Puro (Addgene plasmid #52963) and lentiCas9-Blast (Addgene plasmid #52962) were gifts from Feng Zhang (Sanjana *et al.*). Positive transfected cells, as determined by GFP expression, were selected and enriched with Puromycin (4 µg/µl) and Blasticidin (3.5 µg/µl) for 15 days. NGLY1 KD was confirmed in the cell population by western blotting using the anti-NGLY antibody from Sigma.

Exon 1: gRNA_**2:** CTTGGAGGCCTCCAAAA

Exon 3: gRNA_**1:** TCTGCTACTTCTCTCTA

Single GFP negative cells, negatively selected for loss of the plasmid, were sorted into a 96 well plate and expanded. NGLY1 KD clones were confirmed by Sanger sequencing. Two positively edited clonal lines that had growth rates similar to that of wild type were selected to undergo whole genome sequencing. Libraries were prepared using the Illumina TruSeq protocol following the manufacturer’s directions. After confirmation of the targeted mutations and a lack of evidence for significant off target effects, two lines were chosen for further experiments (see associated genome sequencing data). (Smits, *et al.* 2019)

### Deglycosylation dependent venus reporter cell lines

SS-C-Venus and SS-C-ddVenus reporter sequences were introduced into a modified pRetro-IRES vector (Clontech), expressing mCherry driven by the IRES (Grotzke, Lu, and Cresswell 2013). The pRetroX-IRES-mCherry was created by amplifying the mCherry sequence from pCMV-mCherry-C3 (Clontech) using PJ221 forward primer and PJ222 reverse primer (Table 1). The PCR product was cut with XhoI and BmgBI enzymes and replaced the DsRed sequence which was cut from pRetroX-IRES-dsRed using XhoI enzyme and BmgBI enzyme.

**Table 1:** Primer list

Primer Name	Primer Sequence
PJ221	ACCACGGGGACGTGGTTTTCCTTTGAAAAACACGATGATAATATGGTGAGCAAGGGCGAGGAGG
PJ222	CTTTTATTTTATCCTCGAGCATTCTAAGCTCGTCCATGCCGCCG
PJ181	CCAGTGTGGTGGAATTCTGCAGATATCCAGC
PJ227	GGCATCGCCCTCtcctaccggtGGATCCCGGGTTTAAACGGGCCCCC
PJ219	ccggtaggccGGATCCGCTGATCAGCGGGTTTAAACGGGCCCC

The SS-C-Venus sequence was amplified with the primer PJ181 and PJ219 from plasmid pcDNA-SS-C-Venus, SS-C-ddVenus sequence was amplified with the primer PJ181 and PJ227 from plasmid pcDNA-SS-C-ddVenus (Grotzke, Lu, and Cresswell 2013). Both reporter sequences were cloned into the MCS with Not1 and BamH1 to give rise to pRetro-IRES-mCherry C3-SS-C-Venus and pRetro-IRES-mCherry C3-SS-C-ddVenus.

These plasmids were used to stably integrate the SS-C-Venus and SS-C-ddVenus reporter in the K562 WT cell line, in the NGLY1 KD clone 15 and NGLY1 KD clone 20 by viral transduction according to Clontech’s manual. Positive transduced cells were sorted by mCherry fluorescence.

Prior to analysis, cells were washed twice with PBS + 2% FCS, resuspended in the same, strained through a 40 µM filter, and incubated with 0.5 µg/ml DAPI for live/dead detection on ice until analysis. FACS analysis was carried out on a BD LSR Fortessa measuring Venus fluorescence with 50 mW 488nm excitation paired with a 530/30 nm band pass filter; mCherry fluorescence was measured with 75 mW 561 nm excitation paired with a 610/20 nm band pass filter. Live single cells were selected by exclusion of DAPI positive cells utilizing 20 mW 355 nm excitation paired with a 450/50 nm band pass filter. The geometric mean of the Venus and mCherry signal was used to compute the Venus/mCherry ratio. Analysis was carried out using FlowJo 10.1r7 software, Treestar. Raw .fcs data files and analysis are available upon request and at flowrepository.org: FR-FCM-Z2GJ and FR-FCM-Z2GK.

### RTA∆-V5 reporter cell lines

K562 WT and NGLY1 KD cells were transfected with the pEF/myc/ER- RTA reporter sequence (Kim *et al.* 2006; Tanabe *et al.* 2006; Huang *et al.* 2015) using the Nucleofector 4D device for electroporation according to the manufacturer’s protocol. Cells were selected using G418 treatment at a concentration of 0.8 mg/ml for 14 days.

### RNA extraction, library preparation, and data analysis

RNA was extracted with Trizol according to manufacturer’s protocol used to create RNA-seq libraries using the manufacturers specifications in the Illumina TruSeq Kit protocol.

Transcriptome sequencing was performed on single clones expanded populations of clone 15 and clone 20, each with 2 replicates, as well as 4 wild type cell lines. Raw reads were checked for sequencing quality by *FastQC* (v0.11.5) before alignment to human genome reference GRCh37 using *STAR* (v2.5.1b), with a gene annotation file downloaded from the ENSEMBL database (v75) (Hunt *et al.* 2018; Dobin and Gingeras 2015). The gene expression matrix was counted using *featureCounts* (-p -t exon -Q 255, v1.4.6) and differentially expressed genes were tested with the negative binomial generalized linear model in *DESeq2* (v1.10.1), using Wald test and FDR adjusted p-value < 0.05 (Love, Huber, and Anders 2014). GO enrichment analysis was performed on these significant genes using *Gorilla* (Eden *et al.* 2009). Protein-protein interaction networks were constructed with annotation from the STRING database (Szklarczyk *et al.* 2019). Transcription factors targeting NGLY1 and the significantly deregulated genes were extracted from the Factorbook annotation of the ENCODE project (ENCODE Project Consortium 2012). Compounds targeting NGLY1 were predicted based on the differentially expressed genes in our cells as well as treatment response measurements on multiple cell lines in the CMap/LINCS project (Lamb *et al.* 2006). Data are available at ArrayExpress under the accession E-MTAB-8876.

### Proteomic sample processing and LC-MS/MS analysis

Samples were pre-fractionated with an off-line UltiMate 3000 LPG LC system (Thermo Fisher Scientific), using a basic pH reverse phase separation. Whole cell lysates were fractionated and pooled into 25 fractions. Of these, initially 11 fractions were measured over 120 min on a reverse phase LC gradient, online-injected into a Q Exactive MS instrument (Thermo Fisher Scientific), and data were generated for MS2 applying top10, HCD fragmentation, peptide matching, exclusion of isotopes and dynamic exclusion of precursors. TMT reporter and peptide fragment (amino acid sequence) information was generated in one spectrum and calculated/analyzed/reported by an in-house written software. Database search was done using a Mascot server and the human IPI database. Analysis was carried out on a Q Exactive Plus or Q Exactive HF (both Thermo Fisher Scientific) mass spectrometers coupled to UltiMate 3000 RSLC Nano LC systems (Thermo Fisher Scientific).

Sample preparation for Thermal proteome profiling (TPP) was performed as previously described (Savitski *et al.* 2014). The sample preparation for whole cell expression profiling was performed as follows: Cells were resuspended in lysis buffer (2% SDS, 50 mM Tris-HCl, pH 7.4) and heat treated for 3 min at 95°. Afterward the samples were diluted 1:1 with 50 mM Tris-HCl, pH 7.4 and Benzonase (Sigma-Aldrich E1014) was added at 2 U/µL. Incubation for 30 min at 37° was followed by another addition of Benzonase at 1 U/µL and incubation for 45 min at 37°. The protein extract was cleared from cell debris by centrifugation at 20 000xg for 20 min, the supernatant was snap frozen in liquid nitrogen and stored at -80° until further use.

Gel-based protein separation, peptide labeling, sample pre-fractionation, LC-MS/MS analysis (using Q-Exactive and Fusion Lumos mass spectrometers), protein identification and quantification was performed as previously described (Savitski *et al.* 2018). Data are available upon request and in Supplementary Files 2 and 3.

### Immunoblotting (αNGLY1 and αSNCA)

To obtain total protein lysates, cells were harvested, pelleted and resuspended in protein lysis buffer at ∼30,000 cells/μl, incubated for 30 min at room temperature followed by 95° for 10 minutes. Protein content was assessed by Pierce BCA Protein Assay Kit (Thermo Fisher Scientific) of 1:10 dilutions according to protocol. Protein lysates were heated for 10 min at 70° in NuPage LDS sample buffer, ThermoFisher, supplemented with 4% 2-Mercaptoethanol. 15 - 25 μg total protein was loaded per lane on a NuPAGE 4–12% Bis-Tris Protein Gel (Thermo Fisher Scientific) and protein separation was facilitated in 1x MOPS running buffer for 50 min at 170 V. Precision Plus Protein Dual Color Standards (BioRad) was loaded for size control. Proteins were blotted onto a methanol-activated Immobilon-PSQ PVDF membrane (Millipore, 0.2 μm pore size) in 1x transfer buffer for 90 min at 400 mA, 4°. Subsequently, the membrane was blocked for one hour at room temperature in TBST including 3% (w/v) non-fat dry milk (BioRad), and incubated overnight at 4° with 0.05 μg/ml polyclonal αNGLY1 (1:1000 dilution, Sigma-Aldrich, HPA036825, lot no. B101923) in TBST including 3% (w/v) bovine serum albumin (Sigma-Aldrich). The membrane was extensively washed in TBST and incubated with polyclonal goat-α-rabbit-IgG-HRP (abcam, ab97051) diluted 1:10,000 in TBST/3% milk for an hour at room temperature. After washing the blot was developed using Clarity ECL (BioRad) as substrate and the ChemiDoc Touch (BioRad) for luminescence detection.

To re-probe, antibodies were released from the membrane by 15 min incubation with Restore PLUS Western Blot Stripping Buffer (Thermo Fisher Scientific), after which the membrane was washed extensively with TBST and re-blocked as before.

For the detection of GAPDH, the membrane was incubated for one hour at room temperature with polyclonal αGAPDH (abcam, ab9485, lot no. GR192141-1) diluted 1:2500 in TBST with 3% milk. For the detection of V5, the membrane was incubated over night at 4° with αV5 (Invitrogen, R960-25), 1:1000 in TBST/3% milk. For washing steps, incubation with secondary antibody, repeated washing, and developing were performed as described above.

In the case of αSNCA, the protein gel was run in 1x NuPAGE MES SDS Running Buffer (Thermo Fisher Scientific). After blotting, the proteins were crosslinked to the membrane during 30 min incubation at room temperature in 0.4% paraformaldehyde (Thermo Fisher Scientific) in PBST. Monoclonal antibody against alpha-synuclein (abcam, ab138501, clone MJFR1) was diluted 1:5,000 in PBST/ 3% (w/v) non-fat dry milk.

Buffers and solutions used for immunoblotting included the following: protein lysis buffer (25 mM Tris pH 8.3-8.5, 2% SDS, 20% Glycerol, 1x Complete, EDTA-free (Roche), 0,05 KU Benzonase (Sigma), reducing loading buffer (NuPage LDS sample buffer (4x) ThermoFisher, NP0008, add 4% 2-Mercaptoethanol), MOPS running buffer (50 mM MOPS, 50 mM Tris Base, 0.1% SDS, 1 mM EDTA, pH 7.7), transfer buffer (25 mM Tris Base, 192 mM Glycine, 20% MeOH; TBST: 10 mM Tris, 150 mM NaCl, 0.05% Tween-20), and PBST (137 mM NaCl, 2.7 mM KCl, 10 mM Na2HPO4, 1.8 mM KH2PO4, 0.1% Tween-20).

### Cell culture

Cells were maintained in DMEM (Gibco, 41965-039) with 10% FCS and supplemented with Puromycin (4 µg/µl) and Blasticidin (3.5 µg/µl) at a confluence between 20% and 80%.

### Compounds and plate preparation

Forty-eight compounds were purchased from Selleckchem, and 43 of them solved at 10 mM in DMSO (Table 2). Compounds CP466722 and PIK-90 were solved at 0.75 mM in DMSO, Geneticin and Chloroquine Phosphate were solved at 10 mM in water and NVP-BEZ235 was solved at 10 mM in DMF. 100 µl of the stock compound solutions were manually transferred into a 96 well Matrix tube/rack system for further liquid handling using a robotic system. An 11-fold 1:3-serial dilution of the compounds was prepared from the stock solution in 384-well pp-plates (Greiner 781280) in pure solvent to ensure solubility of the compounds in this step. The peptide Z-VAD-fmk (N-Benzyloxycarbonyl-Val-Ala-Asp(O-Me) fluoromethyl ketone solved at 10 mM in DMSO was diluted similarly and applied as standard to each plate. Three microliter of the serially diluted compounds were transferred to another 384 well pp-plate and 72 µl of cell culture medium (DMEM + 10% FCS) added with a FlexDrop (PerkinElmer) bulk dispenser resulting in 8x concentrated 11-fold 1:3 serial dilution starting at 400 µM (30 µM for the 0.75 mM stock) in medium and 4% solvent. Finally, 5 µl of this compound dilution in medium was transferred to clear 384 well TC-plates (Greiner 781182) and white 384 well TC-plates (PerkinElmer 6007689). The assay plates were sealed with aluminum cover foil and stored at -20°.

**Table 2:** Compound List

Drug	CAS #
Epicatechin	490-46-0
N-acetyl Asparagine	233-716-7
Antimycin A	1397-94-0
Oligomycin A	579-13-5
Rotenone	83-79-4
AZD-8055	1009298-09-2
BMS-536924	468740-43-4
CA-074 methyl ester	147859-80-1
Calpeptin	117591-20-5
Chloroquine Phosphate	50-63-5
CP466722	1080622-86-1
Enzastaurin (LY317615)	170364-57-5
L-690488	142523-14-6
NVP-BEZ235	915019-65-7
PAC-1	315183-21-2
PIK-90	677338-12-4
Temsirolimus	162635-04-3
Aza-cytidine	320-67-2
Ataluren	775304-57-9
Geneticin (G418)	108321-42-2
AG-957	140674-76-6
BO2-inhibits-RAD51	1290541-46-6
Bortezomib	179324-69-7
Carfilzomib	868540-17-4
Cerulenin	17397-89-6
Devazepide	103420-77-5
EMF-bca1-16	1917-65-3
Exemestane	107868-30-4
MLN-2238 (Ixazomib)	1072833-77-2
Moteleukast Sodium	151767-02-1
Nimodipine	66085-59-4
Parthenolide	20554-84-1
Radicicol	12772-57-5
RS-I-002-6 (Isoleucinol)	24629-25-2
Sulfacetamide Sodium	127-56-0
Tanespimycin	75747-14-7
Thapsigargin	67526-95-8
Valproate	99-66-1
VER-155008	1134156-31-2
Arbutin (corrected)	497-76-7
Fludeoxyglucose	29702-43-0
Glucoronamide	3789-97-7
Inosine	292853-81-7
Maltose	133-99-3
Miglustat	72599-27-0
N-Isopropylphtalimide	304-17-6
Sucralose	56038-13-2
Voglibose	83480-29-9

### Screening assay

The assay plates containing the 5 µl of serially diluted compounds were thawed, and 35 µl of reporter cells were added with a MultiDrop bulk dispenser (ThermoFisher), resulting in an 11-fold 1:3 serial dilution starting at 50 µM (3.75 µM for the 0.75 mM stock) in medium containing 0.5% solvent. The assay plates were incubated for 24 hr at 37° before adding 1 µl of 200 µM proteasome inhibitor MG-132 (Sigma M7449), with an additional 6 hr of incubation at 37°. The clear assay plates were used for FACS analysis of the reporter fluorescence. Toxicity of the compounds to the cells was determined in the white cell culture plates by adding 20 µl of ATPLite 1step (PerkinElmer 6016731) with the MultiDrop bulk dispenser and measuring the luminescence in an Envision plate reader (PerkinElmer).

### Bortezomib sensitivity assay

Cells were seeded at a density of 0.5 Mio cells per ml in DMEM and incubated with concentrations between 0 and 400 nM of Bortezomib for 24 hr. The readout was done with ATPLite 1step (PerkinElmer 6016731).

### Proteostat dye assay

The Proteostat Dye (Enzo ENZ-51023-KP050**)** was used according to manufacturer’s protocol. For microscopy, Lab-Tek dishes were coated with poly-L-lysine-hydrobromide (Sigma P6282) before adding cells.

### Analysis by flow cytometry

Analysis was carried out as in Tomlin *et al.* 2017. Briefly, cells were washed twice with PBS + 2% FCS, resuspended in the same, strained through a 40 µM filter, and incubated with 0.5 µg/ml DAPI for live/dead detection on ice until analysis. FACS analysis was carried out on a BD LSR Fortessa measuring Venus fluorescence with 50 mW 488 nm excitation paired with a 530/30 nm band pass filter; mCherry fluorescence was measured with 75 mW 561 nm excitation paired with a 610/20 nm band pass filter. Live single cells were selected by exclusion of DAPI positive cells utilizing 20 mW 355 nm excitation, paired with a 450/50 nm band pass filter. Similar settings were used to analyze the cells on an Intellicyt IQue Screener, exciting DAPI by a 405 nm 50 mW laser. The geometric mean of the Venus and mCherry signal was used to compute the Venus/mCherry ratio. Comparison of treatments between WT and KD (clone 20) cell lines was used to illustrate compound effects. Hits were considered compounds that altered YFP signal by at least 75% percent inhibition compared to control or compounds with multiple concentrations with effects greater than two standard deviations from the plate mean for the experimental cell type.

## Data availability

As mentioned in the above Materials and Methods, cell lines, reagents, and data are available upon request or in the mentioned supplementary files or repositories with the corresponding accession numbers. File S1 contains descriptions of all supplementary material as well as all supplementary figures and discussion. Specifically, proteomics data from whole expression proteomics and TPP is available upon request or in Supplementary Files 2 and 3, raw .fcs data files and analysis are available upon request and at flowrepository.org: FR-FCM-Z2GJ and FR-FCM-Z2GK, and transcript analysis data are available ArrayExpress under the accession E-MTAB-8876. Tables S1 and S2 contain the processed transcriptomic and proteomic data used for the figures in the paper. Supplemental material available at figshare: https://doi.org/10.25387/g3.12014913.

## Results

**Figure 2:**
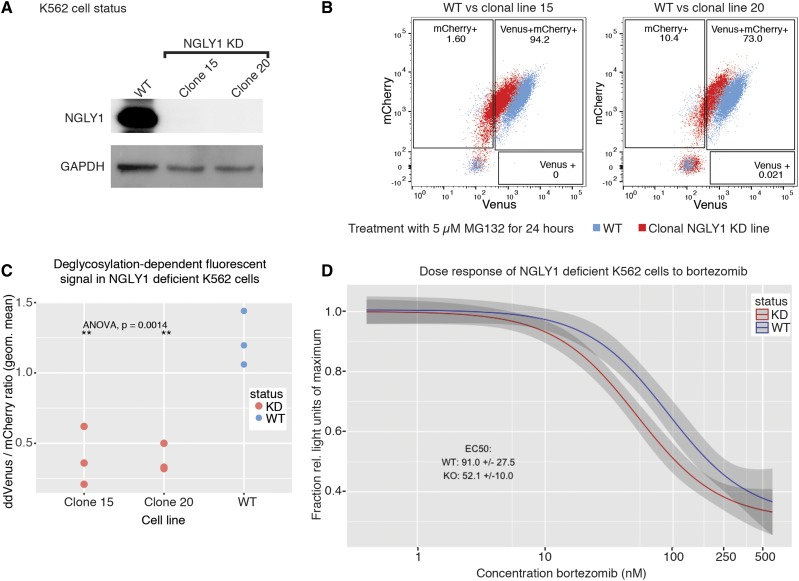
Characterization of NGLY1-deficient K562 lines. (A) Western blot analysis of K562 cell lines used in this paper. (B) Flow readout and gating for the analysis of K562 cell lines used in this paper. Data were used to calculate geometric means for the Venus to mCherry ratio. (C) Average geometric mean of the Venus to mCherry signal for all lines used in the paper. (D) Dose response of NGLY1-deficient K562 lines to Bortezomib. 95% confidence interval shown as shading on the graph.

We used K562 chronic myeloid leukemia cells to study NGLY1 deficiency because these cells are easy to handle, simple to manipulate, and express endogenous NGLY1 at a relatively high level (Uhlen *et al.* 2015). To create NGLY1-deficient cells, we transfected cells with plasmids expressing Cas9 and gRNAs targeting exons 1 and 3 of the *NGLY1* gene (Figure 1A). Successfully transfected cells were selected using GFP expression as a marker, separated by FACS, and grown in clonal populations. These clonal populations were selected for growth rates similar to wild type and lack of NGLY1 expression via western blot analysis (Figure 2A). Targeted mutations were verified using Sanger and whole genome sequencing (WGS, Methods).

To determine the effect of *NGLY1* mutations on NGLY1 activity, we took advantage of the deglycosylation-dependent, NGLY1 activity requiring, Venus (ddVenus) reporter developed in the Cresswell Lab (Grotzke *et al.* 2013). To have a protein expression control, we modified this reporter to contain mCherry protein expressed downstream of the Venus molecule using the EMCV IRES (Figure 1D) (Jang *et al.* 1988). This tandem reporter system was used to measure NGLY1 activity in NGLY1 knock down (KD) cells. Using this system, we found our KD cells achieved a ∼2.5-fold average reduction of ddVenus fluorescence, reflecting a decrease in NGLY1 activity due to the CRISPR-Cas9 mediated mutation of the gene (Figure 2B-C). Upon treatment of our KO cells with Z-VAD-FMK, a known NGLY1 inhibitor, we saw a small reduction in NGLY1 activity. This suggests that our two clonal lines are knock-downs or mutants with some residual activity, not complete knockouts. This is likely due to a small amount of exon skipping that occurs due to the editing in exon 3 (Smits *et al.* 2019). This amount of NGLY1 was not detectable via western blot analysis (Figure 2A).

One of the endogenous targets of NGLY1 in mammalian cells is the transcription factor NFE2L1 (Tomlin *et al.* 2017; Lehrbach *et al.* 2019). NFE2L1 is ubiquitously expressed in human tissues and is involved in the transcriptional control of proteasome bounce back (Y. Zhang and Xiang 2016). It is processed in the ER and translocated to the cytosol, where it is deglycosylated by NGLY1 and cleaved by DDI1. Upon proteasome inhibition, it accumulates and is shuttled to the nucleus to activate proteasome subunit transcription. This mechanism is responsible for the sensitization of NGLY1-deficient systems to proteasome inhibition (Lehrbach and Ruvkun 2016; Tomlin *et al.* 2017; Lehrbach *et al.* 2019). In line with this, we exposed our K562 cell lines to increasing concentrations of Bortezomib (a proteasome inhibitor) and observed that NGLY1 KD K562 cells were ∼twofold more sensitive to treatment than controls (Figure 2D) (Albanell and Adams 2002).

**Figure 3:**
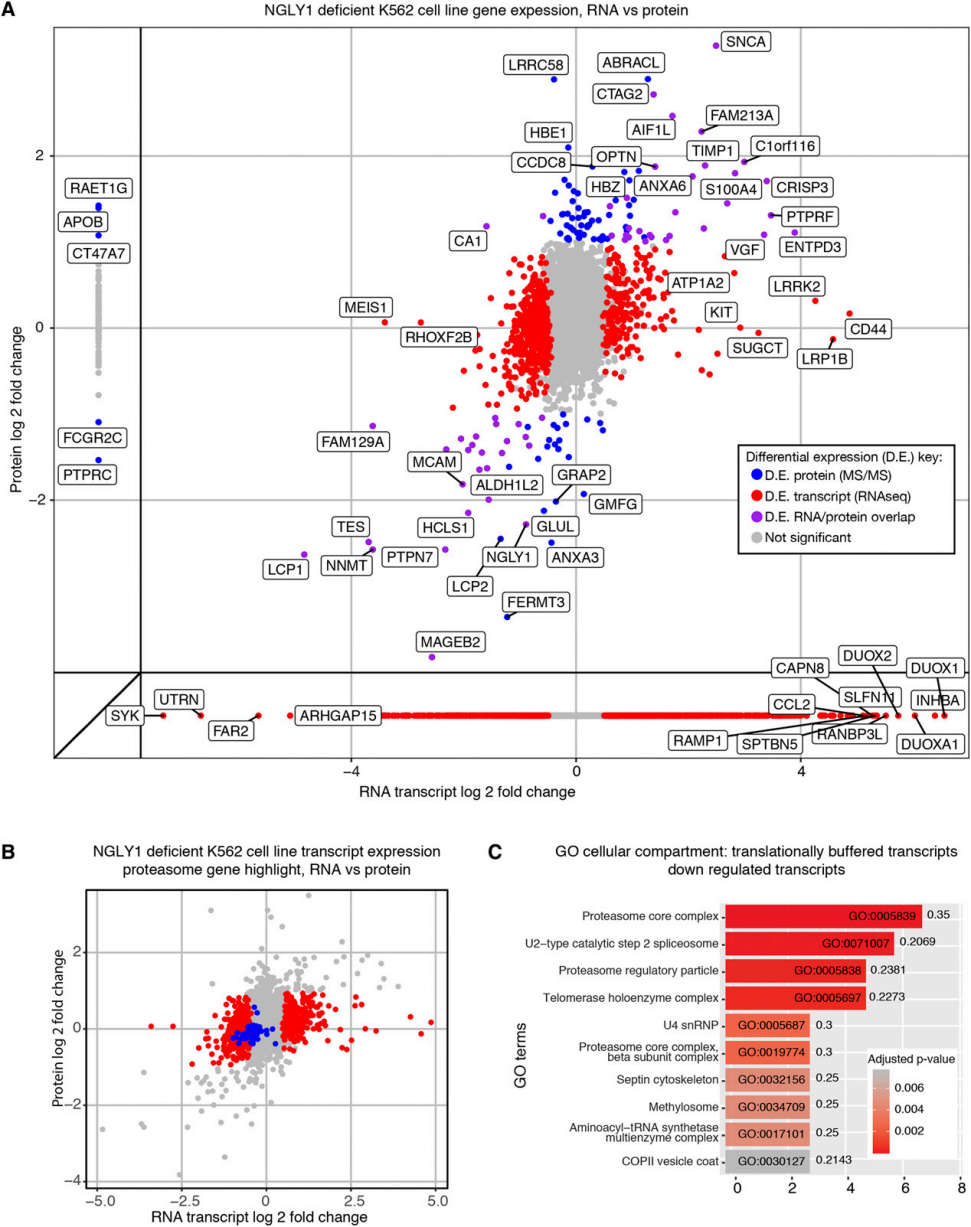
Overlapping correlative analysis of transcripts and proteins detected in NGLY1-deficient K562 cell lines. (A) Fold changes of transcripts and proteins differentially expressed in K562 NGLY1-deficient lines. Colors, as described in the key, correspond to the dataset in which they were found to be differentially expressed. HGNC Gene symbols have been labeled for the most significantly differentially expressed, as determined by a combination of p-value and fold change. (B) Highlight of proteasome subunit gene transcript expression in K562 NGLY1-deficient lines, this gene set (colored in blue) displays a shift toward lower expression in NGLY1-deficient lines. Red points depict the transcripts that were called differentially expressed only on the transcript level. Gray points are all expressed genes. (C) Proteasome subunit genes were identified as significantly enriched downregulated transcripts. X-axis is the number of genes found in the GO category. Numerals after the bar graph represent the ratio of significant genes to total expressed genes in that GO category.

Having established that our NGLY1-deficient system is consistent with previously observed phenotypes, we set out to characterize novel biology associated with NGLY1 deficiency. We harvested whole cell mRNA and protein fractions and performed RNA-seq and LC-MS/MS analyses to determine transcriptional and translational processes that are influenced by NGLY1 (Figure 3A). The ultimate goal of this analysis was to determine pathways that might reveal intermediate phenotypes or traits suitable for screening or prediction of compounds that could influence NGLY1 biology.

We found that there was a broad transcriptional influence, as well as a more subtle proteomic influence, of NGLY1 deficiency on K562 cells. There were ∼1950 transcripts (or 9%) that were differentially expressed (1094 upregulated and 856 downregulated in comparison to WT) compared to 183 proteins (or 3%) that were differentially expressed, Figure 3A, Supplementary Tables S1 and S2). Expression of *NGLY1* was reduced in the KD cell lines approximately twofold on both the RNA and protein level. We first looked for links to previously established NGLY1 related transcriptional activity. Consistent with previous reports, multiple proteasomal genes were moderately downregulated in the K562 cell line (Tomlin *et al.* 2017). This downregulation was not significant for all proteasome genes and was not significant on the protein level (Figure 3B). In general, overlap of our dataset and genes previously shown to be bound by NFE2L1 in K562 cells was low (de Souza 2012). GO analysis did not reveal proteasome subunit genes as enriched, unless we focused our enrichment analysis on genes that were differentially expressed on the transcript-level and not differentially expressed on the protein level (Figure 3C). Further pathway analysis on protein or transcript datasets (gene set enrichment or GO) did not reveal conclusive results (Fig EV1). Multiple growth assays were used to probe possible phenotypes for screening, but no culture conditions used resulted in significantly different growth between WT and KD cell lines, aside from proteasome inhibition.

There were 59 genes whose protein and transcript levels were either both significantly increased, or both significantly decreased in the RNAseq and LC-MS/MS analyses (Figure 3A, points in purple). This set of genes contains NGLY1. We did not find a significant category or gene set that was enriched in this set. SNCA, the gene coding for alpha-synuclein and one of the major proteins involved in aggregate formation in Parkinson’s disease (Cookson 2010), was the most upregulated gene in both our RNA and protein datasets. Two other genes related to alpha-synuclein were found to be upregulated, LRRK2 and FBXO2. Transcript levels of LRRK2, a kinase highly associated with Parkinson’s disease, were also upregulated (Cookson 2010). FBXO2 levels were also upregulated, but to a lower extent. FBXO2 has been implicated in familial forms of Parkinson’s disease.

**Figure 4:**
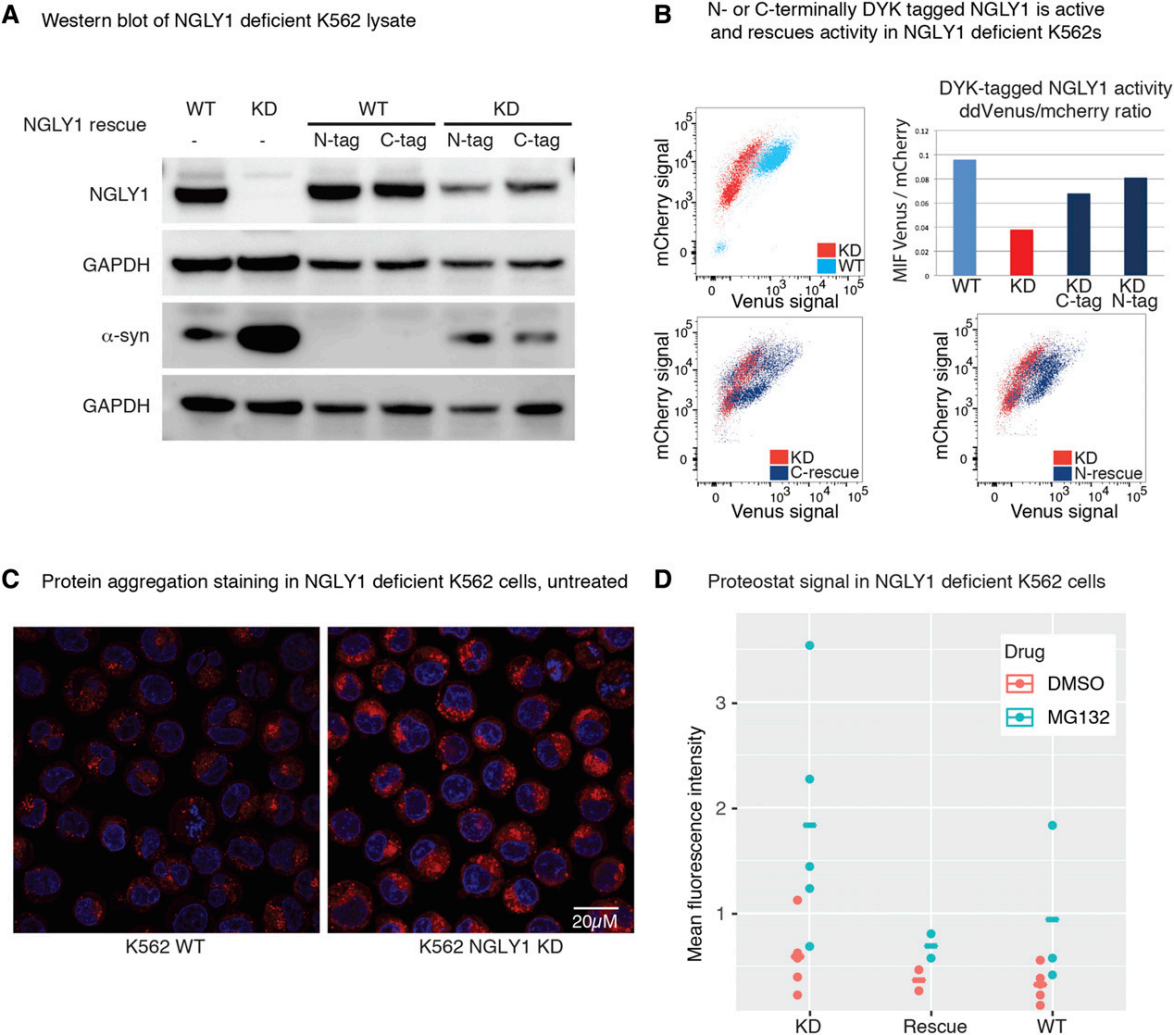
Correction of NGLY1-deficient phenotypes in K562 cell lines by exogenous expression of a DYK-tagged NGLY1 protein. (A) Western blot analysis of NGLY1-deficient K562 cell line's expression of plasmid-based N- or C-terminally tagged NGLY1. Antibodies probed are labeled along the left side of the figure along with corresponding kDa mol. weight markers. The same protein lysates were loaded on two gels. (B) FACS analysis of NGLY1-deficient K562 cell line's expression of plasmid-based N- or C-terminally tagged NGLY1. Results were quantified, averaged, and graphed. (C) Immunofluorescent staining of NGLY1-deficient K562 cell lines with DAPI (blue) and Proteostat (red). (D) Quantification of Proteostat staining of NGLY1-deficient K562 cells by flow cytometry analysis. Rescued lines include both C- and N-terminally tagged NGLY1.

We set out to determine if protein aggregation was observable in our K562 cells. While it has been hypothesized that NGLY1 deficiency causes protein aggregation, there is minimal evidence to support this idea (Need *et al.* 2012; Huang *et al.* 2015). Our finding that SNCA RNA and protein expression is increased in NGLY1 KD cells prompted us to determine if this was directly linked to NGLY1 expression. We tested whether restoration of NGLY1 expression in these cells would reset SNCA expression. To validate that these tagged proteins are active we rescued NGLY1 expression with both C- or N-terminally DYK (FLAG)-tagged recombinant protein and tested activity using the ddVenus FACS assay, finding that both constructs rescue NGLY1 activity (Figure 4A & B). Accordingly, rescue of NGLY1 expression with a C- or N-terminally DYK-tagged recombinant protein decreased SNCA expression back to levels similar to that of WT K562 cells.

Having linked expression of an aggregation-prone protein to NGLY1, we then tested for protein aggregation in the NGLY1-deficient K562 cells using the Proteostat protein aggregation dye (Enzo BioSciences). We found that there was a trend toward increased staining of protein aggregation in K562 NGLY1-deficient cells. This trend could be reversed by over-expression of NGLY1 (Figure 4C and D). This finding suggests that the protein aggregation effect of NGLY1 deficiency can be reversed though rescue of NGLY1 expression. We were unable to show that the aggregates included alpha-synuclein.

To determine if our data could predict putative drug targets that rescue gene expression changes identified or that modulate NGLY1 activity, we input the top 100 significantly differentially regulated transcripts into the CMap database and produced a list of 30 candidate compounds that were found to have a similar or inverse transcriptional profiles upon compound treatment (Lamb *et al.* 2006). We hypothesized that these compounds could have corrective or exacerbatory effects on our K562 NGLY1-deficient system acting through transcriptional mechanisms. To this list of 30 compounds, we added compounds that had previously been predicted to stabilize NGLY1 and compounds that had been used as dietary supplements by NGLY1 patients (Table 2, (Srinivasan *et al.* 2016)).

We assayed these 48 compounds using high-throughput FACS and a plate reader screening with an 11-point dilution curve, testing each compound for its effect on cellular viability (ATPlite) and NGLY1 activity (ddVenus reporter) in duplicate. From our list of 48 compounds, one compound was found to increase ddVenus signal, but this was a false positive due to the intrinsic autofluorescence of the compound matching that of the ddVenus reporter (enzastaurin). No compounds were found that rescued NGLY1 activity (*i.e.*, increased ddVenus signal in the KD cell lines, Supplementary Fig EV3). Proteasome inhibitors increased ddVenus signal slightly but were also toxic, decreasing ATPlite signal considerably. We hypothesized that this was due to increased half-life of ddVenus and the minimal amount of active NGLY1 present due to incomplete KD since deglycosylation is required for fluorescence to accumulate. While our NGLY1 KD cell lines had a small amount of ddVenus signal, no compound mediated decreases in ddVenus signal in the KD cells were identified as significant. 

**Figure 5:**
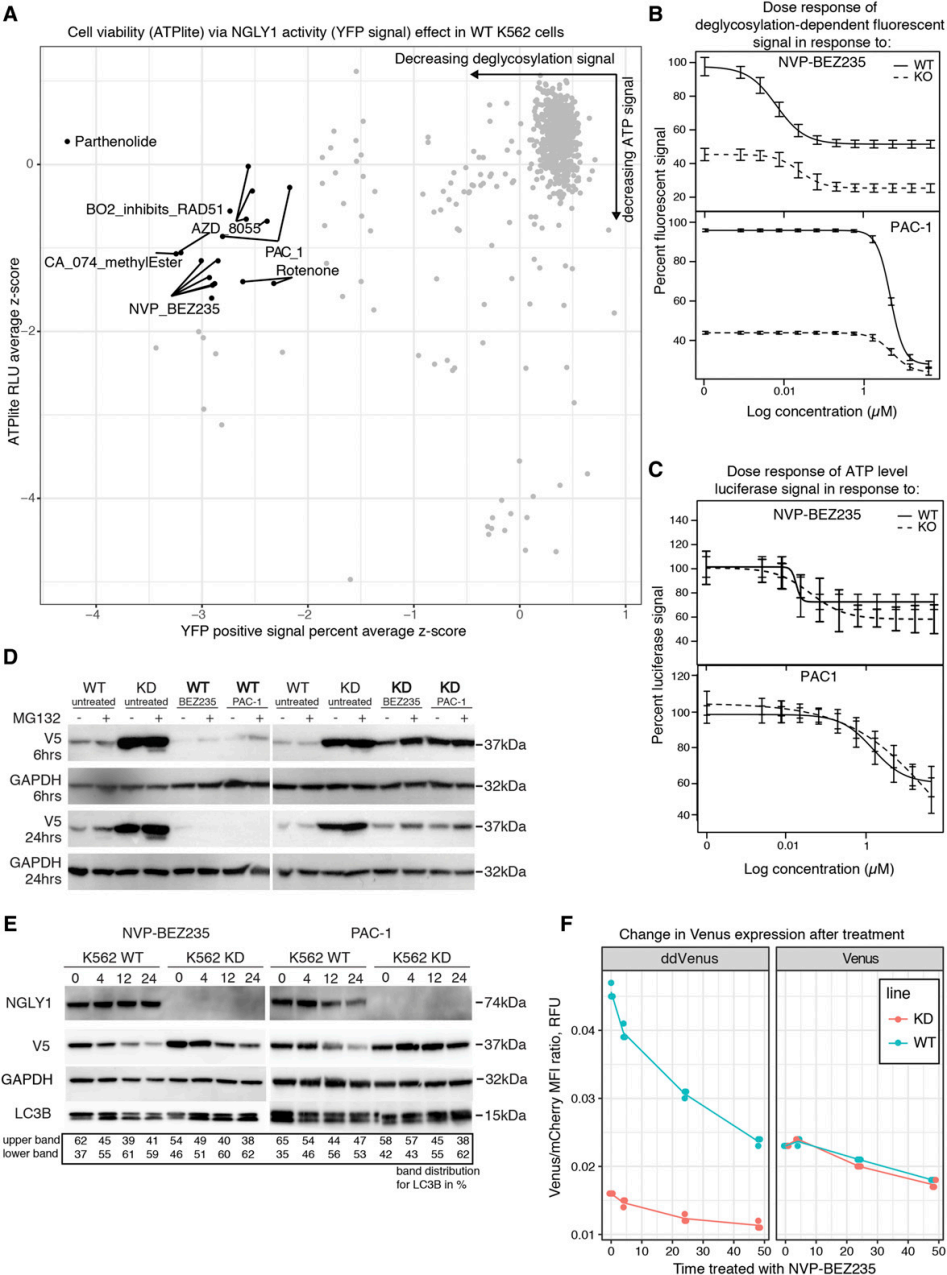
Drug treatment of NGLY1-deficient and WT K562 cell lines. (A) Treatment of WT K562 cells with 48 compounds, plotted by assay and concentration to visualize compounds that were not toxic but still inhibited ddVenus fluorescence. Each point represents a single concentration and are labelled by compound if they decreased ATPlite signal by more than 50% or if they reduced ddVenus signal to the level of the NGLY1 KD line control. (B) Dose response curve for NVP-BEZ235 and PAC-1 treatment of WT and KD NGLY1 K562 cells, exemplative of a positively confirmed hit from the screen. (C) ATP measurement of the dose response curve for NVP-BEZ235 and PAC-1 treatment of WT and KD NGLY1 K562 cells. (D) Western blot analysis of RTA∆-V5 expression levels after NVP-BEZ235 and PAC-1 treatment of WT and KD NGLY1 K562 cells for 5 hr and 24 hr treatment at 15 µM for PAC-1 and 0.5 µM for NVP-BEZ235. Exemplative blot from 3 repeated experiments. (E) Western blot analysis of autophagic flux due to NVP-BEZ235 and PAC-1. Time course treatment of WT K562 cells in the presence of compound. (F) Fluorescent signal of Venus and ddVenus at multiple time points in WT and KD lines.

The assay did identify 6 compounds that seemed to inhibit ddVenus signal. Those 6 compounds decreased ddVenus signal in the WT cell lines by 50% or greater in least two treatment concentrations, maintained cellular viability above 50%, and exhibited some dose response in at least two concentrations (Figure 5A). These compounds were used in small scale dose-response follow up experiments using the same methods and compound concentrations used in the 48 compound screen (Table 3). The follow up ATPlite and ddVenus fluorescence detection experiments were repeated with the 6 ddVenus signal reducing compounds, of those 6, only 2 reduced ddVenus signal below 50% and maintained cellular viability above 50% (Figure 5B and C). The two compounds that validated in both high and low-throughput assays are NVP-BEZ235 (a known PI3K/mTOR dual inhibitor (Maira *et al.* 2008)) and PAC-1 (a Zinc chelating caspase inhibitor (Putt *et al.* 2006)). It should be mentioned that AZD-8055, an mTOR inhibitor, showed ddVenus signal reduction but also reduced cellular viability at similar concentrations (Fig EV4).

**Table 3:** EC50 Values for Compounds in WT and KD cell lines for ATPlite and Deglycosylation assays

Drug	Cell Line	EC50 (µM)	Std. Deviation	Assay	Log EC50 (µM)
AntimycinA	wt	NA	NA	ddVenus	0
AntimycinA	kd	NA	NA	ddVenus	0
Rotenone	wt	2.06	2.28	ddVenus	0.31
Rotenone	kd	0.74	0.76	ddVenus	−0.13
NVP-BEZ235	wt	0.01	0.00	ddVenus	−2.19
NVP-BEZ235	kd	0.03	0.01	ddVenus	−1.60
AZD	wt	0.05	0.02	ddVenus	−1.30
AZD	kd	0.09	0.04	ddVenus	−1.07
PAC1	wt	5.28	1.64	ddVenus	0.72
PAC1	kd	4.14	0.67	ddVenus	0.62
Parthenolide	wt	NA	NA	ddVenus	0.00
Parthenolide	kd	NA	NA	ddVenus	0.00
CA-074	wt	16.83	2.13	ddVenus	1.23
CA-074	kd	2.10	0.68	ddVenus	0.32
AntimycinA	wt	0.00	0.00	ATPlite	−2.54
AntimycinA	kd	0.01	0.02	ATPlite	−2.13
Rotenone	wt	0.39	2.07	ATPlite	−0.40
Rotenone	kd	NA	NA	ATPlite	0.00
NVP-BEZ235	wt	0.017	0.01	ATPlite	−1.75
NVP-BEZ235	kd	0.04	0.03	ATPlite	−1.39
AZD	wt	0.06	0.05	ATPlite	−1.22
AZD	kd	0.12	0.09	ATPlite	−0.92
PAC1	wt	1.63	0.83	ATPlite	0.21
PAC1	kd	NA	NA	ATPlite	0.00
Parthenolide	wt	28.20	57.46	ATPlite	1.45
Parthenolide	kd	8.52	5.60	ATPlite	0.93
CA-074	wt	7.20	3.68	ATPlite	0.86
CA-074	kd	8.63	5.32	ATPlite	0.94

Inhibition of NGLY1 has been shown to adversely impact cancer cell viability, however the ddVenus reporter could be inhibited through alterations to its translation, translocation, or degradation through another means (Tomlin *et al.* 2017; Zolekar *et al.* 2018). To determine if NVP-BEZ235 or PAC-1 were acting directly on NGLY1 or causing indirect effects that could impact ERAD reporters, we used another NGLY1-dependent ERAD substrate, a modified ricin toxin A (RTA∆ tagged with V5) (Grotzke *et al.* 2013; Huang *et al.* 2015). In our hands, expression of the RTA∆-V5 substrate in NGLY1-deficient K562 cells lead to an accumulation of signal by Western blot analysis (Figure 5D), with no consistent discernible change in molecular weight. Our inability to detect a difference in molecular weight in RTA∆ between KD and WT lines is likely due to the activity of ENGase (Huang *et al.* 2015). We treated RTA∆-V5-expressing NGLY1-deficient K562 cells, as well as WT K562 cells, under the hypothesis that treatment with PAC-1 or NVP-BEZ235 would exacerbate this increase in signal through inhibition of NGLY1 or ERAD. However, treatment with NVP-BEZ235 and PAC-1 seemed to cause a decrease in RTA∆-V5 signal (Figure 5D). To determine if this effect correlated with an increase in autophagic flux, as suggested by mTOR/PI3K targeting previously observed in NVP-BEZ235, we looked at the modification of LC3 to LC3-II over time due to compound exposure (Maira *et al.* 2008). We saw a consistent decrease in the level of RTA∆-V5 signal in response to treatment with NVP-BEZ235, but not with PAC-1. The PAC-1 treated V5 signal seems to only visually decrease after 12 hr of treatment in WT cells, or 24 hr in KD cells. This may be indicative of an indirect mechanism. We observed that loss of RTA∆-V5 occurred simultaneously with an increase in the proportion of LC3-II to LC3 upon treatment with NVP-BEZ235, but not PAC-1, suggesting that an increase in autophagy could be responsible for the decrease in RTA∆-V5 upon treatment with NVP-BEZ235 (Figure 5E).

Modulation of mTOR can alter protein synthesis, to make sure that our reporter would not be significantly influenced by treatment with NVP-BEZ235 at the concentrations or time points used in our assays we used a control reporter that is deglycosylation independent (Venus) to look at protein synthesis of the reporter (Ma and Blenis 2009). Both the ddVenus and Venus reporters are upstream of an IRES driving expression of a mCherry protein. After 4 to 24 hr of treatment, there was almost no change or only a slight reduction in the fluorescence signal from the Venus reporter, normalized to the cap-independent mCherry expression (Figure 5F), suggesting that the treatment with NVP-BEZ235 at the concentrations used did not have a large impact on the translation of the reporter and that the changes in abundance observed are not due to translational inhibition. Collectively these data suggest that activation of autophagy clears substrate or protein accumulation due to NGLY1 deficiency.

## Discussion and Conclusions

We generated and profiled a novel NGLY1-deficient cell model that can be used to study NGLY1 biology and used for high throughput screening of NGLY1 activity. We found that this system is consistent with previous observations of NGLY1 deficiency, showing decreased expression of proteasome subunits and increased protein aggregation. Using the expression profile from that system, we identified a compound, Dactosilib (NVP-BEZ235), that induces autophagy and likely does not inhibit NGLY1 activity directly. This increased autophagic flux acts as a compensatory increase in a parallel degradation pathway that can rescue the accumulation of proteins, and possibly NGLY1 dependent substrates. Notably, we also found several proteins implicated in the pathology of Parkinson’s disease that were expressed at higher levels in NGLY1 KD cells.

Dactosilib is currently in clinical trials for cancer (albeit with significant side effects) but has been shown to be protective in mouse models of neurodegenerative disease (Bellozi *et al.* 2016; Wise-Draper *et al.* 2017). The loss of autophagy in dopaminergic neurons is thought to represent a key mechanism in neurodegenerative diseases such as Parkinson’s disease, manifested by increased LRRK2 activity blocking autophagy, and the accumulation of SNCA in Lewy bodies (Schapira and Tolosa 2010). Study of other autophagy inducers, other NGLY1 substrates, other proteins that accumulate in NGLY1 deficient states, and related phenotypes could lead to small compound therapies for NGLY1 deficiency provided that substrate accumulation is found to be causative for clinical phenotypes.

It is unlikely that the increase in NGLY1 substrate degradation through autophagy rescues NFE2L1 processing and sensitivity to proteasome inhibition. If NFE2L1 cannot be post-translationally modified by NGLY1, it will not be able to activate proteasome bounce-back (Lehrbach *et al.* 2019). Removal of proteins that accumulate in NGLY1 deficient cells, like NFE2L1, cannot restore their transcriptional activity, but may be able to help in other ways. There are connections between NGLY1 and mTOR through NFE2L1, but the extent to which an increase in autophagic flux will rescue NGLY1 deficiency is limited by the degradability of accumulated proteins (possibly NGLY1 substrates) in the autophagosome and the likely inability of autophagy to properly post-translationally modify NGLY1 substrates and allow their re-entry into the cytoplasm (Zhang and Manning 2015). Like inhibition of ENGase or activation of NFE2L2, activation of autophagy will likely not rescue all molecular or clinical phenotypes of NGLY1 deficiency, but could only rescue those phenotypes that are related to protein aggregation.

NGLY1 is a zinc-requiring enzyme (Lee *et al.* 2005). Our discovery that NGLY1 is possibly inhibited by zinc chelation treatment with PAC-1 presents another mechanism, like the complementation of proteasome inhibition, for chemical treatment of NGLY1 for cancer. However, due to the odd kinetics of NGLY1 target degradation that we observe in our treatment time course, further validation will be necessary.

The multi-omic phenotypes observed due to the loss of functional NGLY1 were not directly indicative of specific pathways in this study. This may be because NGLY1 seems to act as an early cytosolic step in glycan metabolism and proteostasis of glycoproteins, so may signal multiple pathways through multiple currently unidentified targets (Suzuki *et al.* 2016). Multiple types of stress have been linked to NGLY1-deficient systems: oxidative stress was linked to NGLY1 deficiency through mitochondrial malfunction (Kong *et al.* 2018), proteotoxic stress was linked through abnormal cytosol in patient liver biopsies and reporter protein accumulation in detergent insoluble aggregates (Need *et al.* 2012; Huang *et al.* 2015). Our data show that the canonical stress pathways associated with these systems are not enriched at steady state in the K562 system. However, when perturbed (as observed through proteasome inhibitor treatment) the deficits of the system were revealed, consistent with previous reports that linked NFE2L1 processing to NGLY1 activity (Tomlin *et al.* 2017). An NFE2L1-related signal (downregulation of proteasome subunits) was evident in the set of differentially expressed transcripts that were not differentially regulated on the protein level. This is consistent with sufficient steady state proteostasis that only becomes problematic once the system is challenged. Our data suggest that NGLY1 deficiency will have a larger effect on systems that are more sensitive to proteotoxic, oxidative, or mitochondrial stress.
